# Machine-Learning-Based Late Fusion on Multi-Omics and Multi-Scale Data for Non-Small-Cell Lung Cancer Diagnosis

**DOI:** 10.3390/jpm12040601

**Published:** 2022-04-08

**Authors:** Francisco Carrillo-Perez, Juan Carlos Morales, Daniel Castillo-Secilla, Olivier Gevaert, Ignacio Rojas, Luis Javier Herrera

**Affiliations:** 1 Department of Computer Architecture and Technology, University of Granada, C.I.T.I.C., Periodista Rafael Gómez Montero, 2, 18170 Granada, Spain; juancarlosmv@ugr.es (J.C.M.); irojas@ugr.es (I.R.); jherrera@ugr.es (L.J.H.); 2 Stanford Center for Biomedical Informatics Research (BMIR), Department of Medicine, Stanford University, 1265 Welch Rd, Stanford, CA 94305, USA; ogevaert@stanford.edu; 3 Fujitsu Technology Solutions S.A, CoE Data Intelligence, Camino del Cerro de los Gamos, 1, Pozuelo de Alarcón, 28224 Madrid, Spain; daniel.castillosecilla@fujitsu.com

**Keywords:** NSCLC, machine learning, information fusion, deep learning, personalized medicine, artificial neural networks

## Abstract

Differentiation between the various non-small-cell lung cancer subtypes is crucial for providing an effective treatment to the patient. For this purpose, machine learning techniques have been used in recent years over the available biological data from patients. However, in most cases this problem has been treated using a single-modality approach, not exploring the potential of the multi-scale and multi-omic nature of cancer data for the classification. In this work, we study the fusion of five multi-scale and multi-omic modalities (RNA-Seq, miRNA-Seq, whole-slide imaging, copy number variation, and DNA methylation) by using a late fusion strategy and machine learning techniques. We train an independent machine learning model for each modality and we explore the interactions and gains that can be obtained by fusing their outputs in an increasing manner, by using a novel optimization approach to compute the parameters of the late fusion. The final classification model, using all modalities, obtains an F1 score of 96.81±1.07, an AUC of 0.993±0.004, and an AUPRC of 0.980±0.016, improving those results that each independent model obtains and those presented in the literature for this problem. These obtained results show that leveraging the multi-scale and multi-omic nature of cancer data can enhance the performance of single-modality clinical decision support systems in personalized medicine, consequently improving the diagnosis of the patient.

## 1. Introduction

Lung cancer is one of the deadliest and most common cancer types, accounting for 2.2 million cases and 1.8 million deaths worldwide in 2020 [1]. Non-small-cell lung cancer (NSCLC) is the most prevalent sub-type of lung cancer (LC), representing around 80–85% of the cases [2]. Depending on various factors, two types can be differentiated within NSCLC: lung adenocardinoma (LUAD) and lung squamous cell carcinoma (LUSC). LUAD can be found in the peripheral lung tissue [3], while LUSC is usually centrally located [4,5]. An appropriate identification of the NSCLC lung cancer subtype is critical in the diagnostic process, since therapies differ for LUAD and LUSC [6]. With the advances of computational methods, clinical decision support systems (CDSSs) have been created for cancer detection using biological sources, achieving great results. Among the data sources used in the literature, we have found, for instance, whole-slide imaging (WSI) [7], gene expression data [8], copy number variation (CNV) analysis [9], miRNA expression data [10], or DNA methylation (metDNA) values [11]. By using these modalities independently, an accurate diagnosis can be performed. However, in their inner nature, they provide different biological information that may complement or regulate the information provided by the others. For instance, studies have shown that miRNAs regulate specific genes related to the proliferation of NSCLC [12,13] or methylation and mutation patterns have been predicted using WSI [7,14]. Therefore, exploring whether the fusion of them can provide a more robust diagnosis using computational methods is of great interest for improving the prognosis of the patient.

Information fusion has been a topic of interest in machine learning (ML) in the last decades given the immense amount of heterogeneous information that is being gathered in problems from all areas. The main premise of these methodologies is that the fusion of the information provided by different sources can achieve better results than those obtained by independent classifiers. Three different approaches can be distinguished depending on when the fusion takes place: late, early, and intermediate fusion [15,16,17]. In the late fusion independent classifiers, one for each source of information is trained over the available training data. Then, the outputs produced by these classifiers are fused in order to provide a final prediction, for instance using a weighted sum of the probabilities or by using a majority-voting scheme [18]. By doing so, the mistakes performed by some classifiers can be compensated by the others, improving the final classification. In addition, using a late fusion strategy allows dealing with missing information, which is a very typical setting in biomedical problems.

In this work, we aimed to analyze the fusion of five heterogeneous modalities (WSIs, RNA-Seq, miRNA-Seq, CNV, and metDNA) using a late fusion approach for the LUAD vs. LUSC vs. control classification problem. We evaluated the improvements that can be obtained by fusing information, and the modalities that are crucial to differentiate between the sub-types. In addition, a new late fusion optimization methodology is proposed for this problem, where the weights for the weighted sum of the probabilities are obtained by using a gradient descent approach that takes into account the performance of the fusion model in the classification.

## 2. Related Work

Over the last few years, the potential of ML models using biological data for the diagnosis and prognosis of cancer patients has been shown. Specifically, all the aforementioned biological sources have been used for the creation of CDSS in lung-cancer-related problems.

The use of gene expression data for lung cancer type classification has been explored in the literature in recent years, especially for LUAD given that it is the most frequent NSCLC type. Smolander et al. reached 95.97% accuracy in the LUAD vs. control problem using coding RNA and employing a deep learning model [19]. Likewise, Fan et al. approached the same problem but used support vector machines (SVMs) with a 12-gene signature, obtaining an accuracy of 91% [20]. In addition, some works have been presented for the multiclass classification of lung cancer subtypes. Gonzales et al. presented a model for the classification of small-cell lung cancer (SCLC), LUAD, LUSC, and large-cell lung carcinoma (LCLC) by finding differentially expressed genes (DEGs) and using them as input [21]. By employing RF as the feature selector and k-NN as the classification algorithm they obtained an accuracy value of 88.23%. Castillo-Secilla et al. reached an accuracy of 95.7% using the random forest algorithm in the NSCLC subtype classification task [22]. For the case of miRNA-Seq analysis, some works have been presented in the literature for lung cancer classification. Ye et al. presented a 10-miRNA signature for LUSC vs. control classification, reaching an F1 score of 99.4% [10]. Yang et al. presented an miRNA signature for pathological grading in LUAD [23], reaching an accuracy of 66.19%. In addition, miRNA has shown its potential for pancancer prognosis and treatment recommendation, including LUSC [24]. CNV data have also been used in the literature for lung cancer classification. Qiu et al. presented a CNV signature for LUAD, LUSC, and control classification formed by 33 genes reaching an accuracy of 84% in the validation set [9]. metDNA data have been used in the literature for LUAD vs. control classification, reaching an accuracy of 95.57% by Shen et al. [25]. In addition, the relation of DNA methylation-driven genes with LUSC and LUAD classes was studied by Gevaert et al., finding the clusters of methylation-driven genes that provided clinical implications [26]. Cai et al. tested different feature selection algorithms in combination with different ML algorithms for the task of LUAD vs. LUSC vs. SCLC classification, reaching an accuracy of 86.54% on the task by using a panel of 16 CpGs sites [11].

Deep learning (DL) has shown great potential for computer vision tasks, and therefore, its use combined with WSI has been explored in the literature for NSCLC subtype classification. Coudray et al. presented a convolutional neural network (CNN) using tiles extracted from WSI for LUAD vs. LUSC vs. control classification and mutation prediction, finally reaching an area under the curve (AUC) score of 0.978 in the classification task [7]. By using images manually labeled by experts, Kanavati et al. presented a CNN model using transfer learning for the lung carcinoma vs. control problem, and obtained an AUC score of 0.988 [27]. Finally, other approaches have been presented where deep learning has been combined with more traditional statistics. Graham et al. used tiles extracted from the images and summary statistics to perform the classification between LUAD, control, and LUSC, reaching an accuracy value of 81% [28].

The fusion of the aforementioned sources has been explored in the literature for various lung cancer problems, such as prognosis, grading prediction, or analyzing the relation between them. A deep neural network (DNN) was developed by Lai et al. that combined gene expression and clinical data for prognosis prediction in NSCLC patients [29]. More novel techniques, such as autoencoders, have been explored in the literature for the generation of a feature representation for a later fusion. Cheerla et al. used a deep-learning-based model using miRNA, RNA-Seq, clinical, and WSI data for a pancancer prognosis prediction problem [17]. Similarly, Lee et al. used an autoencoder for obtaining feature representation using mRNA, miRNA, CNV, and metDNA for prognosis prediction [30]. For the problem of grading prediction, Long et al. proposed to use a late fusion methodology along with a gcForest model for predicting the stage of LUAD by fusion RNA-Seq, metDNA, and CNV [31]. The authors reached an F1 score of 88.9% on the task. Finally, in a previous work we showed that the fusion of WSI with RNA-Seq data improved the results obtained by each independent source for the LUAD vs. LUSC vs. control problem [32].

As detailed, previous research has focused on the use of single modalities for the classification, obtaining great results with both molecular and imaging approaches. However, fewer works have been presented in the literature performing a fusion of the information provided by these modalities, missing the opportunity to improve the classification performance and the knowledge acquisition from multiple biological sources. We propose to use the multimodal information to enhance the classification performance for the subtype identification, by leveraging the performance of independent classifiers and exploring the improvements that each source provides. A summary of the different works described for NSCLC classification problems is presented in Table 1.

## 3. Materials and Methods

### 3.1. Data Acquisition and Pre-Processing

In this work we considered four molecular modalities and one imaging modality: RNA-Seq, WSIs, miRNA-Seq, copy number variation and DNA methylation quantification. The data were collected from the Cancer Genome Atlas (TCGA) program [33], which is easily accessible from the GDC portal [34].

Biological and clinical information from 33 different cancer types is contained in TCGA and harmonization of all the samples has been performed by GDC. In most cases for each sample, various modalities are available (e.g., histology imaging, copy number variation, miRNA expression, gene expression, methylation beta values, etc.). Those case IDs used in this work are available in a Github repository (https://github.com/pacocp/multiomic-fusion-NSCLC (accessed on 5 April 2022)). Table 2 shows the number of samples used per class and considered data modality.

To obtain unbiased results avoiding a small test set or data imbalance, a 10-fold cross-validation (10-fold CV) was performed in a stratified and patient-wise way over the whole dataset. By doing this in a stratified way we ensure that we are maintaining the same proportion of classes across the splits, while with a patient-wise method we ensure that the samples from a given patient can only belong to one of the splits in each iteration, be it training or testing. By doing this we are preventing any kind of information leakage between the splits. During each iteration, the training set was used for training the models, to perform the biomarker identification, and for tuning the range of hyperparameters selected for the models, and once they were selected a final performance assessment was performed on the test set. Different strategies were used for the hyperparameter selection depending on the data modality, which will be explained later in the manuscript.

#### 3.1.1. WSI Preprocessing

The Python package openslide was used for the preprocessing of the obtained WSIs. We selected a magnification factor of 20x to obtain images with sufficient resolution for the tile selection process (this magnification factor leaves images with a resolution of ≈10,000 × 10,000 pixels). For the tile selection process, we obtained 512 × 512 non-overlapping tiles of the whole image omitting those where there was a significant amount of background. To test this condition, we computed the mean value for the three color channels and if for the three channels the mean was greater than 220 we discarded that tile, as proposed by other authors in the literature [7]. Otherwise, it was selected for further training. In Table 3 the final distribution of tiles per class can be observed.

#### 3.1.2. Omic Data Preprocessing

To preprocess the RNA-Seq data, the KnowSeq R-Bioc package [22] was used to obtain the DEGs. The *DEGsExtraction* was used over 60,383 genes from the training set in each split, similarly to other works that have been presented in the literature [8,35,36]. As parameters, a $Log_{2}$*fold chain* (LFC) value of 2, a *p*-value of 0.05, and a COV value of 2 were set. For the case of miRNA there is no need to apply a reduction to the number of features since TCGA provides information for 1881 miRNAs.

For metDNA and CNV values, the SciPy ecosystem’s packages were used for the analysis and pre-processing [37]. TCGA contains information from 60,683 genes for CNV data, and 485,577 known CpG sites for metDNA. Both sources contained missing values that were deleted, finally leaving us with 46,585 genes and 365,093 CpG sites for the rest of the pre-processing steps. In order to reduce the number of features, and to investigate the global difference in CNV and metDNA patterns among the three different groups (LUAD, LUSC, and control), a two-tailed t-test was employed (p≤0.001), also using Bonferroni correction as a way to control for the family-wise error rate, as presented by Qui et al. [9]. Those genes and CpG sites that were significantly different in a number of the three two-tailed t-test comparisons (all of them for CNV and two out of the three for metDNA), and for which the difference of the mean was greater or equal to a given threshold (0.1 for CNV and 0.4 for metDNA), were selected in each split.

After performing the aforementioned pre-processing steps, the minimum redundancy maximum relevance (mRMR) algorithm was used over the molecular data for obtaining the most important biomarkers in each modality, by obtaining the mRMR ranking [38]. Taking into account that on every iteration we are using a different training split, this could lead to small variations in the biomarkers obtained each time.

### 3.2. Model Selection and Training

The Resnet-18 architecture was used for WSIs [39], using the pre-trained weights on Imagenet as the starting point [40] and normalizing the tiles using the mean and standard deviation from Imagenet. The last layer was adapted to the set of classes, and only this layer and the last residual block were trained (this last one was fine-tuned). For the selection of hyperparameters, a randomly selected 10% of each training set was used as validation in each split. The network was trained during 25 epochs using an early-stopping methodology where the accuracy in the validation set was monitored, saving the best weights for later use. Adam was used as the optimizer with the following hyperparameters: learning rate value of 1 × 10${}^{-5}$, betas equal to (0.9, 0.999) and epsilon equal to 1 × 10${}^{-8}$, which were selected based on experimentation and results for the hyperparameter validation set. Once the per-tile model was obtained, for classifying a whole slide we followed a majority voting approach, similar to the one presented by Coudray et al. [7], where the final label was the the most predicted class among all slide tiles.

For the rest of the molecular sources different classification algorithms were tested, such as SVMS, k-nearest neighbors, or XGBoost. Finally, SVMs were chosen, since they obtained the best results in the training sets when performing the hyperparameter tuning and they have successfully been used in the literature for cancer classification with good results [8,10,23,35,41]. For tuning the SVM hyperparameters, a grid search CV was used over each training set. The only fixed parameter was the kernel, and we chose the Gaussian radial basis function kernel based on the asymptotic behavior it has [42]. The search range of values for both *C* and γ was $\left[2^{-7},2^{-5},2^{-2},2,2^{4},2^{7}\right]$, and the features used were normalized between −1 and 1.

For implementing the classification models, the Python packages Pytorch [43] and Scikit-Learn [44] were used. In addition, the training of the Resnet-18 architecture was performed in an NVIDIA${}^{\mathrm{TM}}$ RTX 2080 Super graphics processing unit (GPU).

### 3.3. Probability Fusion via Weight-Sum Optimization

There exist two possibilities when applying a late fusion strategy: either to fuse the predictions [45] or the probabilities [46] returned by the classification models. The predictions can be fused by applying a voting scheme, where the most voted class among the different models is the one selected for the fusion model. However, with the probabilities a more fine-grained fusion can be performed, since we have a probability percentage for each class. We chose this last option expecting a better performance based on previous results we have obtained on this problem, when fusing RNA-Seq and WSI [32].

The approach for obtaining the probabilities differs between molecular and imaging modalities. For the molecular modalities, the probability for each class is obtained by using the methodology proposed by Wu et al. [47], based on a coupling method implemented in the SVM classifier that can be found in the Scikit-Learn Python library [44]. For the imaging data, WSIs in this case, we need to manually compute them. Taking into account that we have the predictions for every tile in a given slide, the probabilities are computed as the number of tiles predicted for each class divided by the total number of tiles in the slide (see Equation (1)).

(1) $$P^{{}^{CNN}}\left(x,c_{i}\right)=\frac{\#TilesPredicted\left(x,c_{i}\right)}{\#SlideTiles(x)}$$

where *x* is the sample to be predicted and $c_{i}$ is the given class: LUAD, control, or LUSC.

Different approaches have been proposed in the literature to obtain the weights when there are different models and modalities. For instance, Dong et al. proposed computing the weights based on the performance of the classifiers without any normalization [31], while Meng et al. and Trong et al. proposed normalizing the weights obtained based on the performance by the maximum accuracy or the maximum and the minimum accuracy [48,49]. Other approaches have consisted of simply multiplying the probabilities and the maximum was chosen for the prediction by Depeursinge et al. [46]. In addition, we have previously proposed computing the weights using stratified resampling sets using the performance of each model [32].

One drawback of the aforementioned approaches is that they only take into account the overall performance of the models. However, a classifier can be good at discerning one or various classes but have low overall performance in comparison to the rest of the classifiers. In addition, the weights are computed only once and based on their individual performance, without taking into account how they performed in the classification task when they are fused. In this work, the probabilities from each model and class serve as input to an artificial neural network (ANN), and the weights of the ANN are optimized using a stochastic gradient descent approach. By doing this, we can obtain a weight based on the performance of each classifier for each one of the classes, and where the weights change based on the performance of the fusion model in the classification task.

For the optimization of the weights, an ANN formed by a single linear layer was used in our study. The linear layer has 3 × 5 weights, which could be represented as the following matrix:

(2) $$\left[\begin{array}{ccccc} w_{1,1} & w_{1,2} & w_{1,3} & w_{1,4} & w_{1,5}\\ w_{2,1} & w_{2,2} & w_{2,3} & w_{2,4} & w_{2,5}\\ w_{3,1} & w_{3,2} & w_{3,3} & w_{3,4} & w_{3,5} \end{array}\right]$$

where each row corresponds to a class (LUAD, control, and LUSC) and each column to a data modality (WSI, RNA-Seq, miRNA-Seq, CNV, and metDNA). These weights are randomly initialized but fulfill the condition that the row needs to sum up to one. After each backward pass a softmax function is applied to the weights in order to maintain this condition.

Then, these weights are used to perform a weighted-sum of the probabilities of each class (see Equation (3)), and the final predicted class is the one with the highest probability:

(3) $$\begin{array}{c} P_{Fusion}^{c_{i}}=P_{WSI}^{c_{i}}*w_{i,1}+P_{RNA}^{c_{i}}*w_{i,2}+\\ P_{miRNA}^{c_{i}}*w_{i,3}+P_{CNV}^{c_{i}}*w_{i,4}+P_{DNA}^{c_{i}}*w_{i,5} \end{array}$$

where $c_{i}$ is the class (LUAD, control, or LUSC) and *i* is the index of the class in the weight matrix (see Equation (2)).

Once we have obtained the fused probabilities, the cross entropy loss is used as the loss function in order to optimize the weights for the classification task. By doing this, the optimization allows us to obtain the combination of weights that maximizes the performance in the classification task. The Adam optimizer [50] is used for the optimization once the loss has been computed. A validation set of 10% was selected from each training set, in order to evaluate the performance of the fusion model during the optimization of the weights for 5 epochs.

**Figure 1:**
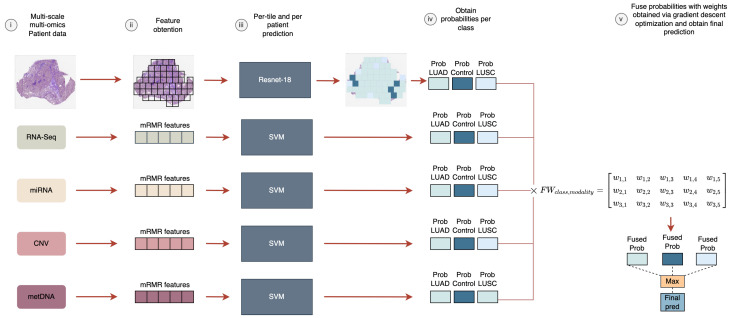
Prediction pipeline for a given sample with multiple modalities. If missing information is present, the probabilities for that modality are zero. (**i**) Multi-scale and multi-omic data available for each sample are obtained. (**ii**) For the imaging modality, non-overlapping tissue tiles of 512 × 512 are obtained. For the molecular modalities, the features are obtained with the aforementioned preprocessing methodology (see Section 3). (**iii**) Probabilities are computed for each modality and class. In the molecular modalities the probabilities are returned by the machine learning model. For the imaging modality, the probabilities are obtained based on the number of tiles predicted per class divided by the total number of tiles. (**iv**) The late fusion model is applied using the previously obtained weights via the gradient optimization, and the final prediction is obtained. (**v**) Fuse probabilities with weights obtained via gradient descent optimization and obtain final prediction.

This methodology allows us to easily deal with missing information, which is crucial when working with biological information given the high cost of performing all the screenings for a patient. If one of the data modalities is missing, its probability for each class will be zero and it would not affect the fusion (see Equation (3)). In Figure 1, an example of the prediction pipeline can be observed.

## 4. Results and Discussion

### 4.1. Performance of Each Data Modality

For the late fusion strategy we need to train independent models using each data modality. In the case of the molecular data, the number of features for each modality was selected based on having the lower number of features that provided the best performance for each independent model, by using the training splits in the 10-fold CV process. Finally, 6 genes were selected for RNA-Seq, 9 miRNA for miRNA-Seq, 12 genes for CNV, and 6 CpGs sites for metDNA data.

The results that were obtained when using each source of information separately can be observed in Table 4, using all the available samples for the three-class classification problem (see Table 2). For the independent models, the higher results for the classification are obtained when using RNA-Seq and metDNA, followed by miRNA-Seq (see Table 4). These results are in accordance with previous studies in NSCLC. Qiu et al. obtained an accuracy of 84% for CNV data [9]. Similarly, the results obtained by Cai et al. (an accuracy of 86.54%) using metDNA are improved by those we have obtained [11]. For the case of WSI, the presented results are very similar to those obtained by Coudray et al. (an AUC of 0.978) and Graham et al. (an accuracy of 81%) [7,28]. For RNA-Seq, Castillo-Secilla et al. reached an accuracy of 94.7% using SVMs, which is similar to our obtained performance [22].

### 4.2. Performance of Late Fusion with Different Number of Sources

Once the models were trained, we tested the different improvements that can be obtained when adding new information, comparing the fusion of the sources in groups of two, three, four, and five. By doing so we were able to see how sources complemented each other in terms of classification performance, and when they improved or worsened it. These results can be observed in Table 4. For the late fusion models we used those samples that the data modalities in use have in common. The number of samples per class are provided as Supplementary Material (see Tables S1 and S2). The confusion matrices for the discussed fusion models are provided as Supplementary Material (see Figure S1).

When fusing two sources, the highest performance in terms of classification metrics was obtained for the fusion of WSI-RNA-Seq, RNA-Seq-miRNA, and RNA-metDNA. Given that RNA-Seq, miRNA-Seq, and metDNA were the ones that achieved the highest performance independently, it was expected that their fusion would provide an increase in the metrics. However, the fusion of WSI and RNA-Seq achieved great results in the classification, even though WSI was not among the sources with the best independent metrics. Therefore, WSI must be improving some of the RNA-Seq predictions, which might be on the wrong side of the prediction border of the probabilities.

Then, we moved to using three sources for the late fusion model. By adding miRNA data to the WSI-RNA-Seq fusion model the results obtained improved (from 94.69±1.80 to 95.69±1.76 in terms of F1 score). The same happened when we included CNV or metDNA in the RNA-Seq-miRNA fusion model. RNA-Seq seems to be the most important source, since it was included in those fusion models with a high performance. In addition, the fusion of RNA-miRNA with other sources improved the classification over using RNA-Seq independently or with other sources.

Finally, we carried out experiments to observe whether there was an improvement in the classification performance when using four or five sources. In this case, the only fusion that improved results over the fusion of three sources, in terms of the F1 score and very similar results in the accuracy metric, was when we fused all the biological sources. However, the improvement was really small and the standard deviation increased (95.69±1.76 for WSI-RNA-Seq-miRNA and 95.82±2.05 for the fusion of all sources). For the rest of the fusion cases, the results obtained are similar to the highest reached when using three sources of information (see Table 4). Therefore, performing more screenings for the patient if you already have the biological sources that provided the best performance when using three sources is not necessary for an accurate diagnosis in this case.

### 4.3. Performance of the Fusion Models with Missing Information

**Figure 2:**
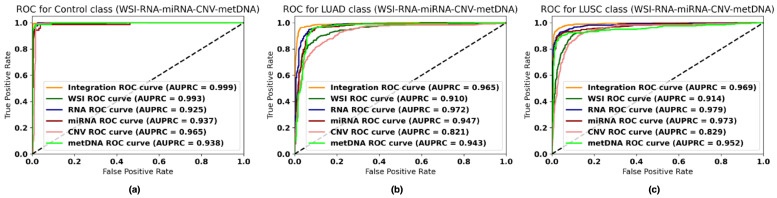
ROC curves for the fusion and individual models over all available samples for each modality. (**a**) ROC curve for LUAD class. (**b**) ROC curve for control class. (**c**) ROC curve for LUSC class.

**Figure 3:**
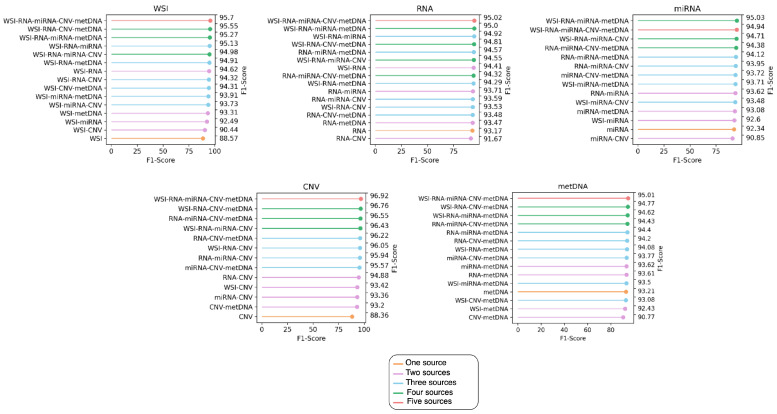
F1 score obtained by each fusion model on the available samples for each modality, without restricting to those in common between the different modalities (see Table 2 to check the number of samples per class). On the left Y-axis the sources used in the integration are shown, while on the right Y-axis the F1 score obtained by each integration can be observed. They are ordered from the highest F1 score to the lowest. metDNA stands for DNA methylation and CNV for copy number variation.

Dealing with missing information is crucial when working with biological sources, given the high cost of some of the screenings. Therefore, we evaluated the effectiveness of the fusion model when some of the modalities were missing. In order to do so, for each fusion model the metrics were computed on all the samples available for the fused modalities, without restricting to those that the modalities have in common. In Figure 2, the receiver operating characteristic (ROC) curves obtained by the fusion model with all modalities can be observed. In Figure 3, the F1 score is presented for each fusion case predicting on all the samples that each modality has (see Table 2).

Except for miRNA-Seq samples, the fusion that achieves the best performance is when fusing the five sources. However, the improvement is small in comparison with using four sources, so not having all of them does not excessively affect the classification performance. Fusing only CNV with metDNA RNA or miRNA-Seq worsens the performance in comparison to the usage of them independently, which could be due to the imbalance in the classes (Table 2). The combination of metDNA and WSI also performs poorly, maybe due to the fact that it has been shown in the literature that WSI reflects information about the methylation patterns of human tumors [14], and therefore, they might not be complementing each other. However, in most cases, including additional information improves the results that can be obtained by each independent source.

The final results obtained when fusing all the data sources is an F1 score of 96.82±1.07, an accuracy of 96.81±1.07, an AUC of 0.993±0.004, and an AUPRC of 0.980±0.016. These results improved those aforementioned and also reduced the standard deviation obtained across the splits. The ROC curves (Figure 2) obtained show the performance of each individual modality and the fusion model over all the available samples for each one (all the samples in the case of the fusion model). The fusion model outperforms each modality for the three classes, showing the potential of using all the information. In addition, the fusion model reduces the number of misclassified samples for all sources, representing a reduction in the diagnosis error rate rate up to ≈8.6% in the best case and ≈1.6% in the worst case (see Table 5). The confusion matrix obtained over the whole dataset is presented in the Supplementary Material (see Figure S2) along with the weights obtained for each modality in the fusion (see Supplementary Material Table S4).

### 4.4. Comparison with Previous Work

The majority of the works presented in the literature for NSCLC subtypes and control classification have focused on using a single data modality and mainly a bi-class classification problem. Our fusion model outperforms or reaches the same results obtained by those works where an LUAD vs. LUSC vs. control classification has been presented, and a summary is presented in Table 6. The fusion of information improves the results that Qiu et al. obtained for CNV data (an accuracy of 84%) [9]. Similarly, the results obtained by Cai et al. using metDNA are also improved (they obtained an accuracy of 86.54%) while reducing the number of CpG site signatures [11] and similar results were obtained compared to those presented by Castillo-Secilla et al. using RNA-Seq (accuracy of 95.7%) [22]. For the case of WSI, the fusion also improved the results presented in the literature by Coudray et al. [7] (an AUC of 0.978) reaching an AUC of 0.991. In the case of multi-omic fusion, we have not found works presenting methods for the NSCLC subtypes and control classification. However, in other NSCLC-related problems the fusion of information has presented an enhancement in the performance. Cheerla et al. [17] showed that by fusing clinical, miRNA, and WSI data, the performance was improved in LUAD prognosis prediction. Similarly, Lee et al. [30] improved the prognosis prediction by fusing the information of four sources (RNA-Seq, miRNA, CNV, and metDNA) over each independent one. This same behavior was observed in our case for these sources.

When it comes to the relations between data modalities, our results highlight previously reported patterns. It has been presented in the literature that WSI can be used to predict mutation patterns or gene expression levels [7,51], so the information provided may be completed with the one presented in RNA-Seq data. Similarly, when fusing three data modalities it was shown that including miRNA-Seq in the RNA-Seq-WSI fusion model improved the classification performance. miRNAs regulate specific genes related to the proliferation of NSCLC [12,13], and therefore, might be complementing the information provided by RNA-Seq and WSI.

## 5. Conclusions

In this paper, we demonstrated the usefulness of fusing heterogeneous sources of biological information for NSCLC subtypes and control classification. In addition, we proposed a new optimization methodology for weighting the classifiers in a late fusion strategy, effectively dealing with missing information and reaching good performance in the classification.

The fusion of the information outperformed the use of each independent source for the classification. Independently, RNA-Seq and metDNA achieved the highest performance in the classification. When performing the fusion, RNA-Seq is crucial for the classification problem and the addition of miRNA-Seq in combination with another data modality improved the obtained results. The best results were obtained when fusing the five sources of information reaching an F1 score of 96.82±1.07 when classifying all the available samples from all sources. However, there was not a huge increase in comparison with using three or four sources. The obtained results also highlight other reported patterns in the literature between data modalities that should be further studied. In addition, the methodology effectively deals with missing information, which is mandatory given that not all screenings are always performed to a patient. The presented methodology can be used in any diagnosing problem where heterogeneous sources of information are available, and it can be extended to any number of data sources.

As future work we would like to test the generalization capabilities of the proposed methodology for the classification of other cancer types or in other diagnosis-related problems and evaluate whether the relations found between the different modalities apply to these other problems.

## Figures and Tables

**Table 1:** Summary of works in the literature for different NSCLC classification problems. SVM: support vector machine; DNN: deep neural network; RF: random forest; CNN: convolutional neural network; k-NN: k-nearest neighbor; Acc.: accuracy; AUC: area under the curve.

	Modalities	Problem	Model	Metrics	Results
Smolander et al. [19]	RNA-Seq	LUAD vs. control	DNN	Acc.	95.97%
Fan et al. [20]	RNA-Seq	LUAD vs. control	SVM	Acc.	91%
Gonzales et al. [21]	Microarray	SCLC vs. LUAD vs. LUSC vs. LCLC	k-NN	Acc.	91%
Castillo-Secilla et al. [22]	RNA-Seq	LUAD vs. control vs. LUSC	RF	Acc.	95.7%
Ye et al. [10]	miRNA-Seq	LUSC vs. control	SVM	F1 score	99.4%
Qiu et al. [9]	CNV	LUAD vs. control vs. LUSC	EN-PLS-NB	Acc.	84%
Shen et al. [25]	metDNA	LUAD vs. control	RF	Acc.	95.57%
Cai et al. [11]	metDNA	LUAD vs. LUSC vs. SCLC	Ensemble	Acc.	86.54%
Coudray et al. [7]	WSI	LUAD vs. control vs. LUSC	CNN	AUC	0.978
Kanavati et al. [27]	WSI	Lung carcinoma vs. control	CNN	AUC	0.988
Graham et al. [28]	WSI	LUAD vs. control vs. LUSC	CNN	Acc.	81%

**Table 2:** Number of samples per class for each data modality.

	WSI	RNA-Seq	miRNA	CNV	metDNA
LUAD	495	457	413	465	431
Control	419	44	71	919	71
LUSC	506	479	420	472	381
Total	1420	980	904	1856	883

**Table 3:** Number of tiles obtained from the WSI per class.

	# Tiles
LUAD	100,841
Control	62,715
LUSC	92,584
Total	256,140

**Table 4:** Results obtained in the 10-fold CV by each single modality and multimodal fusion of the modalities in their common samples (see Supplementary Material Tables S1–S3). For the case of four- and five-modality fusion, AUC is omitted given the low number of control samples. The X marks the modalities that are used in each case.

WSI	RNA-Seq	miRNA	CNV	metDNA	Acc. (Std)	F1 score (Std)	AUC (Std)	AUPRC (Std)
X					88.56 (2.34)	88.57 (2.36)	0.965 (0.003)	0.940 (0.014)
	X				93.16 (1.87)	93.17 (1.82)	0.987 (0.007)	0.973 (0.028)
		X			92.31 (2.69)	92.34 (2.65)	0.976 (0.013)	0.961 (0.023)
			X		88.36 (1.34)	88.36 (1.34)	0.954 (0.009)	0.879 (0.025)
				X	93.21 (1.84)	93.19 (1.87)	0.972 (0.016)	0.957 (0.030)
X	X				94.65 (1.80)	94.69 (1.80)	0.991 (0.004)	0.979 (0.032)
X		X			92.59 (2.57)	92.60 (2.56)	0.987 (0.006)	0.982 (0.009)
X			X		90.26 (1.98)	90.20 (1.92)	0.974 (0.010)	0.962 (0.016)
X				X	92.79 (1.77)	92.80 (1.78)	0.983 (0.009)	0.979 (0.012)
	X	X			94.55 (1.83)	94.74 (1.70)	0.988 (0.007)	0.980 (0.017)
	X		X		91.81 (2.34)	92.12 (2.36)	0.978 (0.006)	0.953 (0.050)
	X			X	94.33 (1.81)	94.33 (1.79)	0.991 (0.007)	0.989 (0.009)
		X	X		91.00 (1.97)	91.36 (1.82)	0.973 (0.009)	0.944 (0.048)
		X		X	93.84 (2.88)	93.85 (2.88)	0.979 (0.015)	0.980 (0.015)
			X	X	90.15 (3.09)	90.28 (3.04)	0.968 (0.010)	0.947 (0.033)
X	X	X			95.55 (1.78)	95.69 (1.76)	0.985 (0.008)	0.990 (0.005)
X	X		X		93.99 (1.47)	94.00 (1.41)	0.982 (0.022)	0.974 (0.041)
X	X			X	94.70 (2.11)	94.73 (2.10)	0.987 (0.010)	0.990 (0.007)
X		X	X		93.84 (2.05)	93.97 (2.03)	0.974 (0.030)	0.977 (0.016)
X		X		X	94.23 (2.55)	94.23 (2.54)	0.975 (0.022)	0.986 (0.008)
X			X	X	93.50 (2.98)	93.52 (2.97)	0.981 (0.009)	0.978 (0.012)
	X	X	X		94.79 (1.76)	95.10 (1.72)	0.938 (0.059)	0.963 (0.050)
	X	X		X	95.05 (2.05)	95.10 (2.01)	0.967 (0.027)	0.989 (0.009)
	X		X	X	94.11 (1.76)	94.20 (1.74)	0.977 (0.012)	0.981 (0.010)
		X	X	X	94.11 (2.92)	94.36 (2.70)	0.975 (0.005)	0.966 (0.023)
X	X	X	X		95.22 (2.13)	95.47 (2.01)	-	0.987 (0.007)
X	X	X		X	95.53 (2.09)	95.62 (2.04)	-	0.989 (0.007)
X	X		X	X	95.22 (2.10)	95.30 (2.05)	-	0.986 (0.009)
X		X	X	X	94.71 (2.29)	94.9 (2.20)	-	0.978 (0.013)
	X	X	X	X	94.86 (2.19)	95.14 (2.06)	-	0.981 (0.010)
X	X	X	X	X	95.53 (2.20)	95.82 (2.05)	-	0.983 (0.012)

**Table 5:** Correct and misclassified samples over the whole dataset for each data type and the fusion model using all modalities. RNA, CNV, and metDNA stand for RNA-Seq, copy number variation, and DNA methylation, respectively.

	WSI	RNA	miRNA	CNV	metDNA
Correct	1232	913	834	1636	821
Misclassified	159	67	70	220	62
Fusion					
Correct	1328	929	857	1796	838
Misclassified	63	51	47	60	45
Absolute difference in misclassified error rate (#samples (%))	96 (6.5%)	16 (1.6%)	23 (2.6%)	160 (8.6%)	17 (2%)

**Table 6:** Comparison of our fusion results with the available literature for LUAD vs. control vs. LUSC. The results of the fusion model are on those available samples for the studied modality. Unfortunately, a direct comparison of fusion methods cannot be performed given the lack of literature for this specific problem. The best results for each case are highlighted in bold.

	Modality	Metric	Score
Qui et al. [9]	CNV	Acc.	84%
Ours	CNV	Acc.	**96.93%**
Cai et al. [11]	metDNA	Acc.	86.54%
Ours	metDNA	Acc.	**95.01%**
Cai et al. [11]	metDNA	F1 score	74.55%
Ours	metDNA	F1 score	**95.01%**
Castillo-Secilla et al. [22]	RNA-Seq	Acc.	**95.7%**
Ours	RNA-Seq	Acc.	95%
Castillo-Secilla et al. [22]	RNA-Seq	F1 score	**95.4%**
Ours	RNA-Seq	F1 score	95.02%
Coudray et al. [7]	WSI	AUC	0.978
Ours	WSI	AUC	**0.991**
Graham et al. [28]	WSI	Acc.	81%
Ours	WSI	Acc.	**95.70%**

## Data Availability

The case IDs used in this work and the code, which will be released after acceptance of this work, can be found in the following Github repository: https://github.com/pacocp/multiomic-fusion-NSCLC (accessed on 5 April 2022).

## Supplementary Materials

The following are available online at https://www.mdpi.com/article/10.3390/jpm12040601/s1, Figure S1: Confusion matrices obtained for different fusion models in the samples that the modalities have in common. WSI stands for Whole-Slide-Imaging, CNV stands for Copy Number Variation, and DNA for DNA Methylation, Figure S2: Confusion matrix for the fusion model using all modalities on all the available samples, without restricting to those that the modalities have in common (see Table 2 in main text). WSI stands for Whole-Slide-Imaging, CNV stands for Copy Number Variation, and metDNA for DNA Methylation, Table S1: Number of samples in common per class when we integrate two sources of information. WSI stands for Whole-Slide-Imaging, CNV stands for Copy Number Variation, and metDNA for DNA Methylation, Table S2: Number of samples in common per class when we integrate three sources of information. WSI stands for Whole-Slide-Imaging, CNV stands for Copy Number Variation, and metDNA for DNA Methylation, Table S3: Number of samples in common per class when we integrate four and five sources of information. WSI stands for Whole-Slide-Imaging, CNV stands for Copy Number Variation, and metDNA for DNA Methylation, Table S4: Ranges for the weights obtained for the five sources fusion and class in the 10 Fold-CV. The range presents the minimum and maximum values obtained with the optimization across the splits. WSI stands for Whole-Slide-Imaging, CNV stands for Copy Number Variation, and metDNA for DNA Methylation.

## Abbreviations

The following abbreviations are used in this manuscript:

LUAD	Lung adenocarcinoma
LUSC	Lung squamous cell carcinoma
NSCLC	Non-small-cell lung cancer
SVM	Support vector machines
CNN	Convolutional neural network
CNV	Copy number variation
metDNA	DNA methylation
WSI	Whole-slide imaging
CDSS	Clinical decision support system
RF	Random forest
mRMR	Minimum redundancy maximum relevance
AUC	Area under the curve
ANN	Artificial neural network
