# Supplementary material for: Machine-Learning-Based Late Fusion on Multi-Omics and Multi-Scale Data for Non-Small-Cell Lung Cancer Diagnosis
Source: J Pers Med. 2022 Apr 8;12(4):601. doi: 10.3390/jpm12040601 (PMC9025878; doi:10.3390/jpm12040601)
Supplement: Supplementary file 1 [file jpm-12-00601-s001.zip › jpm-1655720-Supplementary.pdf]

Supplementary Material

Francisco Carrillo-Perez, Juan Carlos Morales, Daniel Castillo-Secilla,
Olivier Gevaert, Ignacio Rojas and Luis Javier Herrera

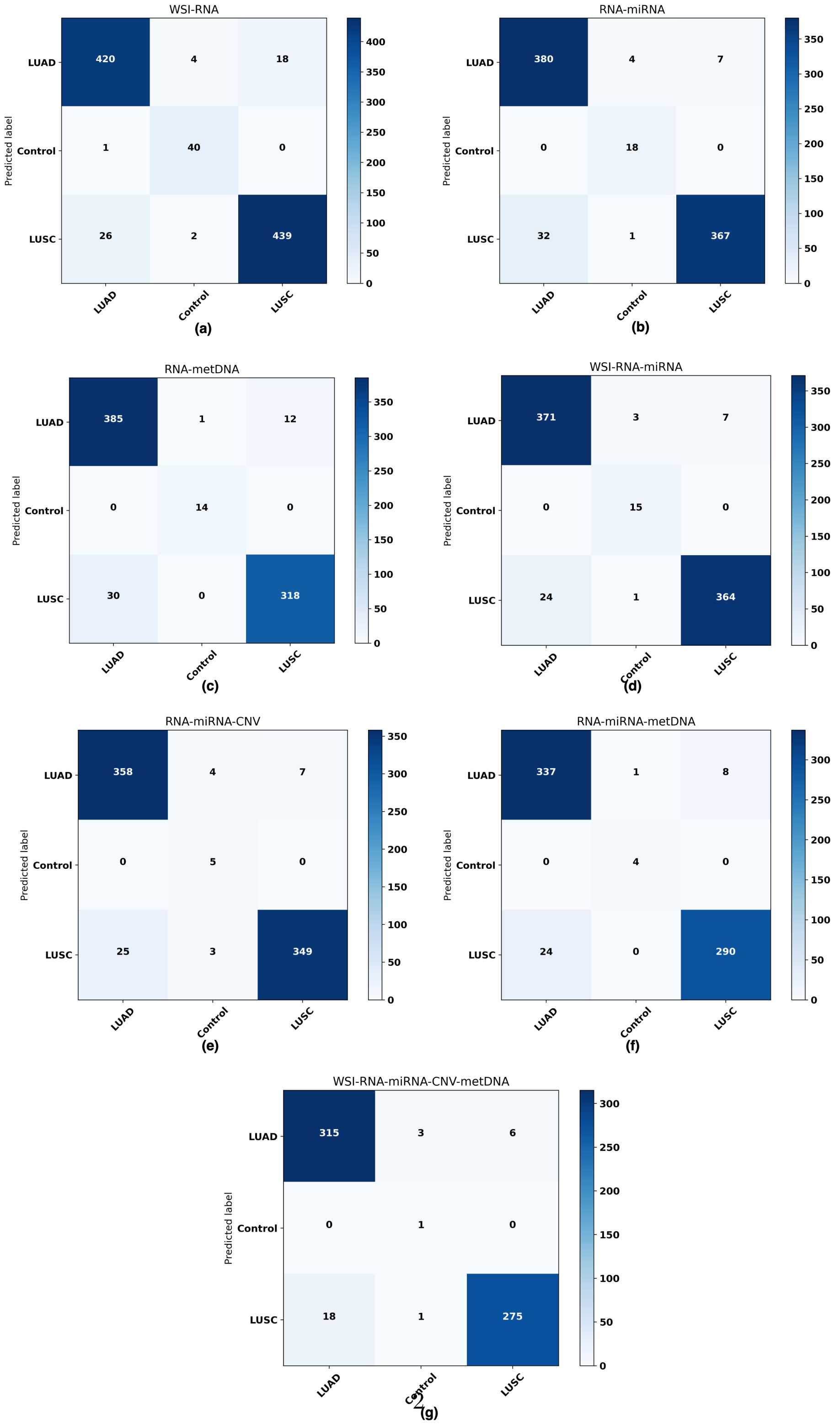

Figure S1: Confusion matrices obtained for different fusion models in the samples that the modalities have in common. WSI stands for Whole-Slide-Imaging, CNV stands for Copy Number Variation, and DNA for DNA Methylation.

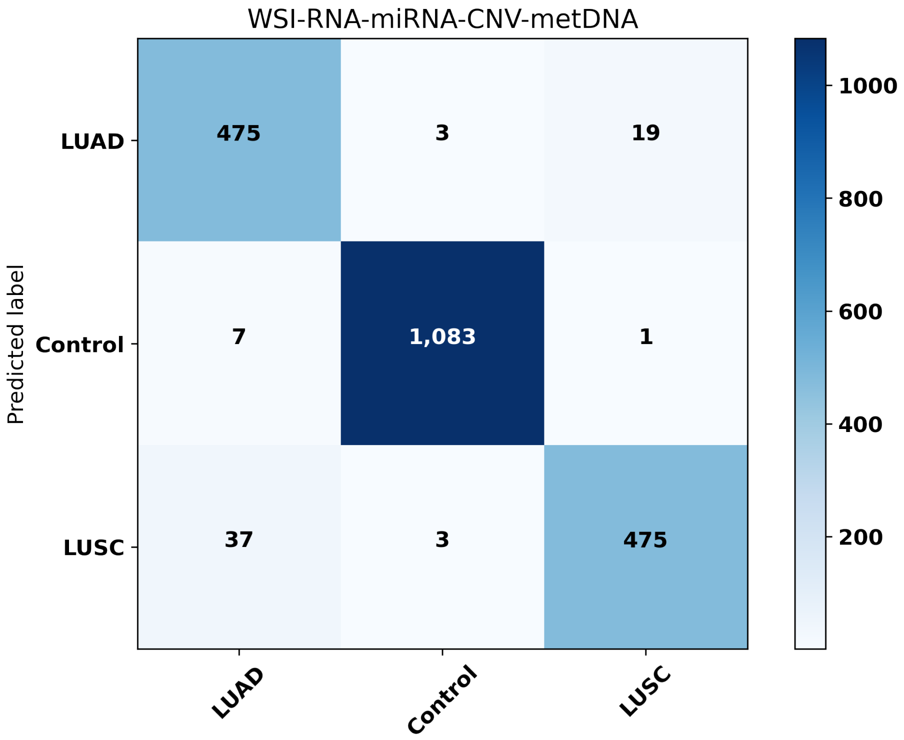

Figure S2: Confusion matrix for the fusion model using all modalities on all the available samples, without restricting to those that the modalities have in common (see Table 2 in main text). WSI stands for Whole-Slide-Imaging, CNV stands for Copy Number Variation, and metDNA for DNA Methylation.

Table S1: Number of samples in common per class when we integrate two sources of information. WSI stands for Whole-Slide-Imaging, CNV stands for Copy Number Variation, and metDNA for DNA Methylation.

**Fusion**	LUAD	Control	LUSC
WSI-RNA	442	41	467
WSI-miRNA	402	68	405
WSI-CNV	451	251	457
WSI-metDNA	415	71	356
RNA-miRNA	391	18	400
RNA-CNV	433	23	448
RNA-metDNA	398	14	348
miRNA-CNV	385	20	397
miRNA-metDNA	367	9	334
CNV-metDNA	441	52	361

Table S2: Number of samples in common per class when we integrate three sources of information. WSI stands for Whole-Slide-Imaging, CNV stands for Copy Number Variation, and metDNA for DNA Methylation.

**Fusion**	LUAD	Control	LUSC
WSI-RNA-miRNA	381	15	389
WSI-RNA-CNV	419	23	437
WSI-RNA-metDNA	383	14	336
WSI-miRNA-CNV	376	20	383
WSI-miRNA-metDNA	356	9	319
WSI-CNV-metDNA	397	52	346
RNA-miRNA-CNV	369	5	377
RNA-miRNA-metDNA	346	4	314
RNA-CNV-metDNA	383	10	337
miRNA-CNV-metDNA	349	2	324

Table S3: Number of samples in common per class when we integrate four and five sources of information. WSI stands for Whole-Slide-Imaging, CNV stands for Copy Number Variation, and metDNA for DNA Methylation.

**Fusion**	LUAD	Control	LUSC
WSI-RNA-miRNA-CNV	360	5	367
WSI-RNA-miRNA-metDNA	336	4	303
WSI-RNA-CNV-metDNA	369	10	326
RNA-miRNA-CNV-metDNA	333	1	304
WSI-RNA-miRNA-CNV-metDNA	324	1	294

Table S4: Ranges for the weights obtained for the five sources fusion and class in the 10 Fold-CV. The range presents the minimum and maximum values obtained with the optimization across the splits. WSI stands for Whole-Slide-Imaging, CNV stands for Copy Number Variation, and metDNA for DNA Methylation.

**Fusion**		LUAD	Control	LUSC
WSI-RNA-miRNA-CNV-metDNA	WSI	[0.20, 0.33]	[0.21, 0.30]	[0.20, 0.28]
	RNA	[0.17, 0.30]	[0.11, 0.16]	[0.17, 0.22]
	miRNA	[0.15, 0.20]	[0.14, 0.20]	[0.17, 0.21]
	CNV	[0.16, 0.21]	[0.27, 0.34]	[0.17, 0.24]
	metDNA	[0.16, 0.21]	[0.11, 0.17]	[0.14, 0.22]
